# Hydrochemical Characteristics, Controlling Factors, and High Nitrate Hazards of Shallow Groundwater in an Urban Area of Southwestern China

**DOI:** 10.3390/toxics13060516

**Published:** 2025-06-19

**Authors:** Chang Yang, Si Chen, Jianhui Dong, Yunhui Zhang, Yangshuang Wang, Wulue Kang, Xingjun Zhang, Yuanyi Liang, Dunkai Fu, Yuting Yan, Shiming Yang

**Affiliations:** 1 Chongqing Institute of Geology and Mineral Resources, Chongqing 401120, China; 2 Chongqing Huadi Resources Environment Technology Co., Ltd., Chongqing 401120, China; 3 Sichuan Provincial Engineering Research Center of City Solid Waste Energy and Building Materials Conversion and Utilization Technology, Chengdu 610106, China; 4 Yibin Research Institute, Southwest Jiaotong University, Yibin 644000, China; 5 Faculty of Geosciences and Environmental Engineering, Southwest Jiaotong University, Chengdu 611756, China; 6 Sichuan Province Engineering Technology Research Center of Ecological Mitigation of Geohazards in Tibet Plateau Transportation Corridors, Chengdu 611756, China

**Keywords:** nitrate, groundwater, land use, stable isotopes, drinking water quality

## Abstract

Groundwater nitrate (NO_3_^−^) contamination has emerged as a critical global environmental issue, posing serious human health risks. This study systematically investigated the hydrochemical processes, sources of NO_3_^−^ pollution, the impact of land use on NO_3_^−^ pollution, and drinking water safety in an urban area of southwestern China. Thirty-one groundwater samples were collected and analyzed for major hydrochemical parameters and dual isotopic composition of NO_3_^−^ (δ^15^N-NO_3_^−^ and δ^18^O-NO_3_^−^). The groundwater samples were characterized by neutral to slightly alkaline nature, and were dominated by the Ca-HCO_3_ type. Hydrochemical analysis revealed that water–rock interactions, including carbonate dissolution, silicate weathering, and cation exchange, were the primary natural processes controlling hydrochemistry. Additionally, anthropogenic influences have significantly altered NO_3_^−^ concentration. A total of 19.35% of the samples exceeded the Chinese guideline limit of 20 mg/L for NO_3_^−^. Isotopic evidence suggested that primary sources of NO_3_^−^ in groundwater include NH_4_^+^-based fertilizer, soil organic nitrogen, sewage, and manure. Spatial distribution maps indicated that the spatial distribution of NO_3_^−^ concentration correlated strongly with land use types. Elevated NO_3_^−^ levels were observed in areas dominated by agriculture and artificial surfaces, while lower concentrations were associated with grass-covered ridge areas. The unabsorbed NH_4_^+^ from nitrogen fertilizer entered groundwater along with precipitation and irrigation water infiltration. The direct discharge of domestic sewage and improper disposal of livestock manure contributed substantially to NO_3_^−^ pollution. The nitrogen fixation capacity of the grassland ecosystem led to a relatively low NO_3_^−^ concentration in the ridge region. Despite elevated NO_3_^−^ and F^−^ concentrations, the entropy weighted water quality index (EWQI) indicated that all groundwater samples were suitable for drinking. This study provides valuable insights into NO_3_^−^ source identification and hydrochemical processes across varying land-use types.

## 1. Introduction

Groundwater serves as a vital global source of drinking water, yet its quality is facing escalating threats, especially in agricultural regions [1,2,3]. A survey demonstrates that approximately one-third of the world’s population relies on groundwater as their primary drinking water supply, with agrarian areas exhibiting significantly higher contamination risks [4]. Among various pollutants, nitrate contamination is the most widespread issue linked to intensive agricultural processes [5,6,7]. For instance, previous studies revealed severe nitrate pollution hotspots: over 30 regions in Africa, 20 areas in Asia, and nine regions in Europe had average groundwater nitrate concentrations exceeding the WHO safety guideline (50 mg/L) [8], while in China, approximately 20% of shallow groundwater samples surpassed the national standard (20 mg/L) [9]. The primary driver of this contamination is the excessive use of nitrogen-based fertilizers and manure in agriculture [10,11,12], which disrupts natural hydrogeochemical cycles. Moreover, long-term exposure to nitrate-rich groundwater greatly threatens human health [13,14,15], such as gastric cancer. Despite these documented risks, systematic investigations into pollution sources and hydrochemical controls remain limited in many high-risk agricultural zones [16]. Therefore, comprehensive assessments of groundwater chemistry and anthropogenic influences are urgently needed to guide targeted water resource protection policies.

Previous studies have utilized conventional hydrochemical analytical approaches, including major ion ratio analysis and basic graphical methods, including Piper and Gibbs diagrams, to investigate groundwater contamination in agricultural areas [17,18,19]. These methodologies have indicated that nitrate contamination predominantly stems from chemical fertilizers, manure, and wastewater [20,21,22]. For example, Yang et al. [23] demonstrated that excessive nitrogen fertilizer application leads to nitrate release into soils, consequently elevating nitrate concentrations in the groundwater of China’s Ordos Basin. Jin et al. [24] identified manure as a significant contributor to elevated nitrate levels in groundwater systems. On the other hand, to assess the implications of nitrate-contaminated groundwater, researchers have conducted comprehensive water quality evaluations [25,26,27,28]. Various quantitative methods have been employed to determine drinking water suitability, including the single-factor index [29], Nemerow index [30,31], and water quality index (WQI) [32,33,34]. The single-factor index offers a rapid and straightforward assessment of parameter exceedances, whereas the Nemerow index provides a holistic evaluation by integrating multiple pollutant concentrations [35,36,37]. The WQI, meanwhile, simplifies complex water quality data into an interpretable numerical score, facilitating informed decision-making [38,39,40]. Collectively, these methods yield critical insights for groundwater quality protection and remediation strategies.

While these conventional approaches have proven effective in elucidating nitrate pollution sources and assessing groundwater suitability, they exhibit notable methodological limitations [41,42,43]. A primary constraint lies in the frequent ambiguity of source identification due to overlapping ionic ratios among different nitrogen sources [44,45,46]. Furthermore, most existing studies inadequately consider potential hydrochemical processes that may modify or obscure source signatures. Similarly, current water quality assessment methods face critical shortcomings, particularly in their inability to quantitatively evaluate drinking water suitability or provide entirely objective assessment outcomes [47,48]. However, recent methodological advancements demonstrate promising solutions to these limitations through the integration of dual nitrate isotopes (δ^15^N and δ^18^O) and the entropy weighted water quality index (EWQI) [49,50,51]. The isotopic approach leverages distinct source-specific signatures, minimizing uncertainties associated with mixed pollution sources. Concurrently, the EWQI enhances objectivity by quantifying assessment results and applying information entropy theory to derive statistically robust weighting coefficients [52,53].

Anthropogenic activities have influenced the groundwater chemistry to some extent [54]. Different land use types reflect different natural environments and human influences, which significantly impact groundwater quality [55]. Several studies have demonstrated that nitrate pollution strongly correlates with land use types [56]. For example, Iqbal found that land use types, particularly cropland and rural areas, strongly correlate with nitrate concentration [57]. Choi found that 23%, 43%, and 67% of samples in the cropping area, the cropping–livestock farming complex area, and the residential area exceeded the national nitrate concentration standard [56]. Exploring the impact of different land use types on nitrate concentrations is crucial for the sustainable agricultural management and precise pollution control.

As a vital area for economic development, the southwestern region of China has seen increasingly prominent nitrate pollution in groundwater due to intensified agricultural activities and accelerated urbanization in recent years [58,59]. Consequently, research on this region has grown progressively more comprehensive and in-depth. However, systematic studies on groundwater in the southwestern region remain insufficient, leaving residents in some areas poorly informed about local groundwater conditions. This situation contradicts China’s commitment to sustainable development and significantly hinders regional economic growth. The study area is located in southwestern China, where agricultural development and drinking water quality are critical for economic development. Nevertheless, research on hydrochemical evolution and groundwater quality remains scarce. To address this limitation, this study employs hydrochemical analysis, ion ratios, and isotopic characteristics to identify nitrate sources, while using the EWQI to assess drinking water quality. Accordingly, the objectives of this study are as follows: (1) determine the general characteristics and spatial distribution of hydrochemical parameters in the study area, (2) analyze the sources of major ions and nitrate in groundwater, (3) identify the impact of land use type on NO_3_^−^ pollution, and (4) evaluate groundwater quality for drinking and its spatial distribution in the study area. The findings of this research will provide valuable insights for groundwater management worldwide.

## 2. Study Area

**Figure 1:**
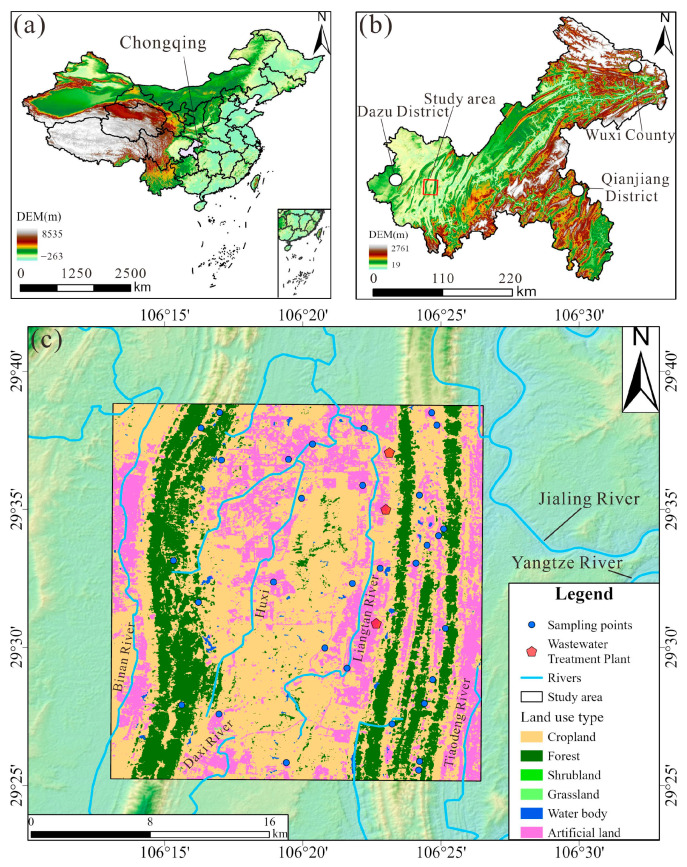
The location of the groundwater samples: (**a**) location of Chongqing City; (**b**) location of the study area in Chongqing City; (**c**) land use map of the study area and the location map of the groundwater samples.

The study area is located in the southwest part of Chongqing city, southwestern China, spanning from 106°13′ to 106°26′ E and 29°25′ to 29°38′ N (Figure 1). The geomorphology is characterized by parallel ridges and valleys trending NE-NNE. The area experiences a humid subtropical monsoon climate, with an average annual rainfall of approximately 1188 mm, and an average annual temperature of 18.63 °C.

Geologically, the valley regions are primarily underlain by the Suining Formation (J_3_sn) and the Shaximiao Formation (J_2_s) of the Jurassic strata. The left-side ridges are mainly composed of the Feixianguan Formation (T_1_f), Jialingjiang Formation (T_1_j), and Leikoupo Formation (T_2_l) of the Triassic strata. In contrast, the right-side ridges are composed of the Longxing Formation (P_2_l), Changxing Formation (P_2_c), and Maokou Formation (P_1_m) of the Permian strata. The valley regions are dominated by sandstone, mudstone, and shale, whereas the ridge regions are predominantly composed of dolomite and limestone.

Groundwater in the study area occurs mainly in two forms: (1) bedrock fracture water within the weathering zones of red beds, and (2) fracture water within carbonate rocks. Bedrock fracture water is mainly found in valley regions, typically at depths less than 20 m. Carbonate fracture water is distributed in a banded pattern along the axes of anticlines. Groundwater is primarily recharged by precipitation and is discharged through springs and underground rivers.

Land use in the study area includes six categories: cropland, forest, shrubland, grassland, water body, and artificial land (Figure 1c). Forested areas are mainly distributed along ridge regions, while cultivated land is concentrated in the valley areas between ridges. Artificial surfaces refer to impervious land covers such as asphalt, concrete, and building structures, which are primarily focused near ridges and in the northern section of the central valley. The study area boasts abundant industrial resources, featuring two major industrial parks. It enjoys unique port advantages with multiple open platforms. Additionally, the area is home to several universities and hospitals. Notably, there are three wastewater treatment plants situated near the right ridge.

## 3. Materials and Methods

### 3.1. Field Sampling and Laboratory Analysis

A total of 31 groundwater samples were collected from wells at depths of 20–50 m across the study area. The sampling aquifer in the valley was the weathered fissure aquifer of the bedrock in red beds, while the ridge groundwater was taken from the karst aquifer of carbonate rocks. Before sampling, each well was purged by continuous pumping for 10–15 min to ensure the collection of fresh groundwater. Temperature, pH, and electrical conductivity (EC) were measured using a portable multi-parameter water quality analyzer (Multi3 630 IDS). Groundwater samples were filtered through 0.22 μm membrane filters and stored in 1 L high-density polyethylene (HDPE) bottles pre-rinsed three times with deionized water. Immediately after filtration, concentrated nitric acid (HNO_3_, 1:1) was added dropwise until the sample pH was below 2 to preserve the samples. Samples for NO_3_^−^ dual isotopes (δ^15^N–NO_3_^−^ and δ^18^O–NO_3_^−^) were collected in 500 mL HDPE bottles, also pre-rinsed with deionized water. All samples were sealed with paraffin wax immediately after collection and stored in a portable insulated cooler at 0–4 °C. Samples were then transported to the Sichuan Geological Survey Institute for laboratory analysis.

Hydrochemical parameters were analyzed: the total hardness (TH) was detected by the EDTA volumetric method, and the total dissolved solids (TDS) was detected by the gravimetric method. Major cations (K^+^, Na^+^, Ca^2+^, and Mg^2+^) were analyzed by the inductively coupled plasma atomic emission spectrometry method (ICP-AES). Cl^−^ and SO_4_^2−^ were detected by the ion chromatography method. HCO_3_^−^ was measured by the HCl titration method. NO_3_^−^ was estimated by disulfophenol spectrophotometry. F^−^ was determined by the ion-selective electrode method. The dual isotopes of NO_3_^−^ (δ^15^N–NO_3_^−^ and δ^18^O–NO_3_^−^) were analyzed using isotope ratio mass spectrometry (IRMS), with an analytical precision of ±0.5‰. To ensure analytical reliability, 20% of the total samples were randomly selected for duplicate analysis as part of the quality assurance (QA) protocol. Certified reference materials (CRMs) and blank samples were included in each batch for quality control (QC).

Cation balance error (*CBE*) calculations showed that all samples had errors within ±5%, indicating acceptable ionic balance and overall data reliability (Equation (1)).

(1) $$CBE=\frac{\left|cation-anion\right|}{cation+anion}$$

### 3.2. Entropy Water Quality Index (EWQI)

The entropy water quality index (EWQI) is a multi-parameter comprehensive evaluation method based on information entropy theory. Compared with conventional evaluation methods, the EWQI eliminates the subjectivity of manually assigned weights. As a result, it provides a more objective, systematic, and comprehensive assessment of water quality status. The calculation procedure of the EWQI involves the following steps:

(1) Scale the hydrochemical data (Equations (2) and (3)).

(2) $$X=\left[\begin{array}{cccc} x_{11} & x_{12} & \ldots & x_{1n}\\ x_{21} & x_{22} & \ldots & x_{2n}\\ \vdots & \vdots & \ddots & \vdots \\ x_{m1} & x_{m2} & \ldots & x_{mn} \end{array}\right]$$

(3) $$Y=\{\begin{array}{c} \frac{x_{ij}-{\left(x_{ij}\right)}_{min}}{{\left(x_{ij}\right)}_{max}-{\left(x_{ij}\right)}_{min}}+0.0001\\ \frac{{\left(x_{ij}\right)}_{max}-x_{ij}}{{\left(x_{ij}\right)}_{max}-{\left(x_{ij}\right)}_{min}}+0.0001 \end{array}$$

(2) Calculate the weight of each hydrochemical parameter (Equations (4) and (5)).

(4) $$P_{ij}=\frac{y_{ij}}{\sum_{i=1}^{m}y_{ij}}$$

(5) $$e_{j}=-\frac{1}{lnm}\sum_{i=1}^{m}P_{ij}lnP_{ij}$$

(6) $$w_{j}=\frac{1-e_{j}}{\sum_{j=1}^{n}1-e_{j}}$$

(3) Determine the quantitative rating scale *q* (Equations (7) and (8)).

(7) $$q_{j}=\frac{C_{j}}{S_{j}}\times 100$$

(8) $$q_{pH}=\{\begin{array}{cc} \frac{C_{pH}-7}{8.5-7}\times 100 & C_{pH}>7\\ \frac{7-C_{pH}}{7-6.5}\times 100 & C_{pH}<7 \end{array}$$

(4) Calculate the EWQI value based on *q* and *w* (Equation (9)).

(9) $$\mathrm{EWQI}=\sum_{j=1}^{n}\left(w_{j}\times q_{j}\right)$$

## 4. Results and Discussion

### 4.1. Hydrochemical Characteristics of Hydrochemical Parameters

**Figure 2:**
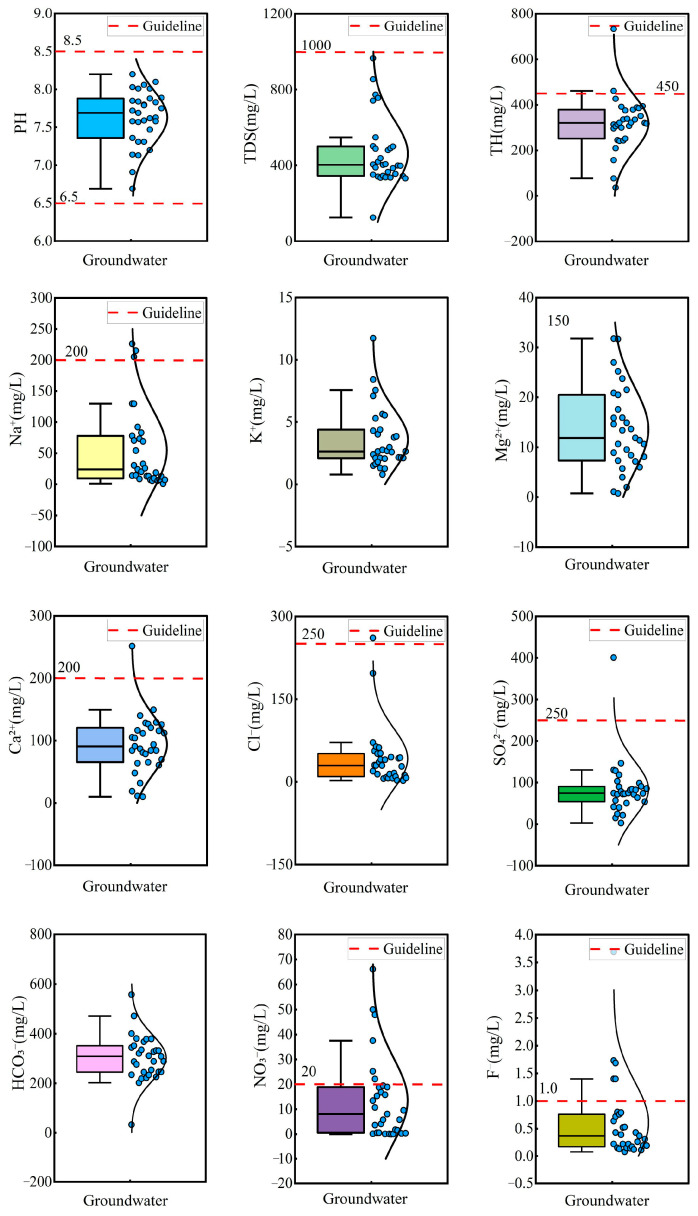
Box plots of hydrochemical parameters in groundwater.

**Figure 3:**
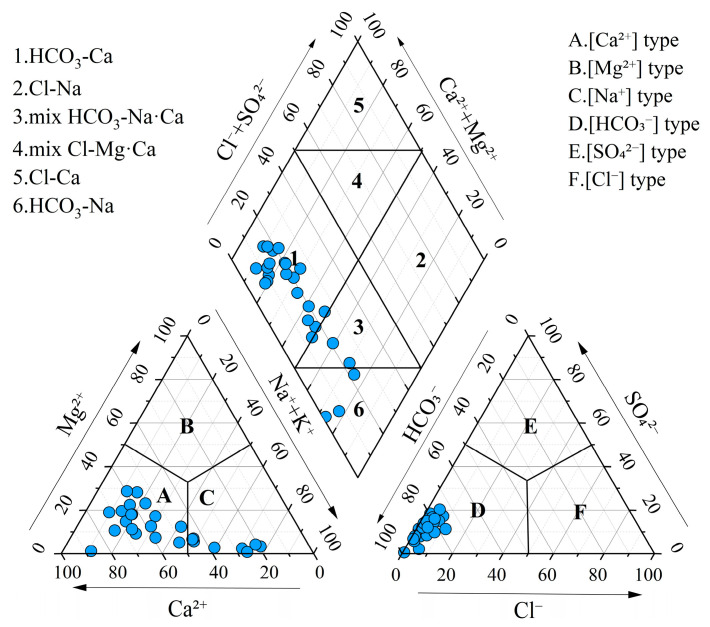
Piper diagram for hydrochemical types of groundwater.

Statistical analysis and box plots of hydrochemical parameters in groundwater are summarized in Table 1 and Figure 2. The pH ranged from 6.69 to 8.20, with a mean value of 7.63, indicating that the groundwater was neutral to slightly alkaline. Total hardness (TH) varied significantly, ranging from 36.03 mg/L to 733.16 mg/L, with a mean concentration of 318.56 mg/L and a standard deviation (SD) of 118.06 mg/L. Total dissolved solids (TDS) ranged from 125.00 mg/L to 965.55 mg/L, with a mean of 461.54 mg/L and a standard deviation of 176.54 mg/L, classifying the groundwater as fresh water and slightly hard water. Piper diagrams are widely used for the classification of hydrochemical facies [60]. Most groundwater samples were categorized as Ca-HCO_3_ type, while the remaining samples were classified as mixed Na·Ca-HCO_3_ type and Na-HCO_3_ type (Figure 3). This suggested the groundwater was undergoing a hydrochemical evolution from Ca–HCO_3_ type to Na–HCO_3_ type.

The mean concentrations of cations followed the descending order: Ca^2+^ (93.47 mg/L) > Na^+^ (54.73 mg/L) > Mg^2+^ (13.60 mg/L) > K^+^ (3.59 mg/L), while the anion concentrations were ranked as: HCO_3_^−^ (303.18 mg/L) > SO_4_^2−^ (84.09 mg/L) > Cl^−^ (42.12 mg/L) >NO_3_^−^ (13.56 mg/L) > F^−^ (0.61 mg/L). According to Chinese drinking water standards [61], 3.22% of groundwater exceeded the permission limit for Na^+^, SO_4_^2−^_,_ and Cl^−^, indicating minor contamination from these ions. Notably, 19.35% and 16.13% of groundwater exceeded the permission limit for NO_3_^−^ and F^−^, implying potential pollution from NO_3_^−^ and F^−^. The coefficient of variation (CV) was used to assess the degree of dispersion of hydrochemical parameters. The CV of Cl^−^, NO_3_^−^, F^−^, and Na^+^ exceeded 100%, reflecting significant variability and suggesting strong anthropogenic or environmental influences on their distribution.

### 4.2. Spatial Distribution Characteristics of Groundwater

**Figure 4:**
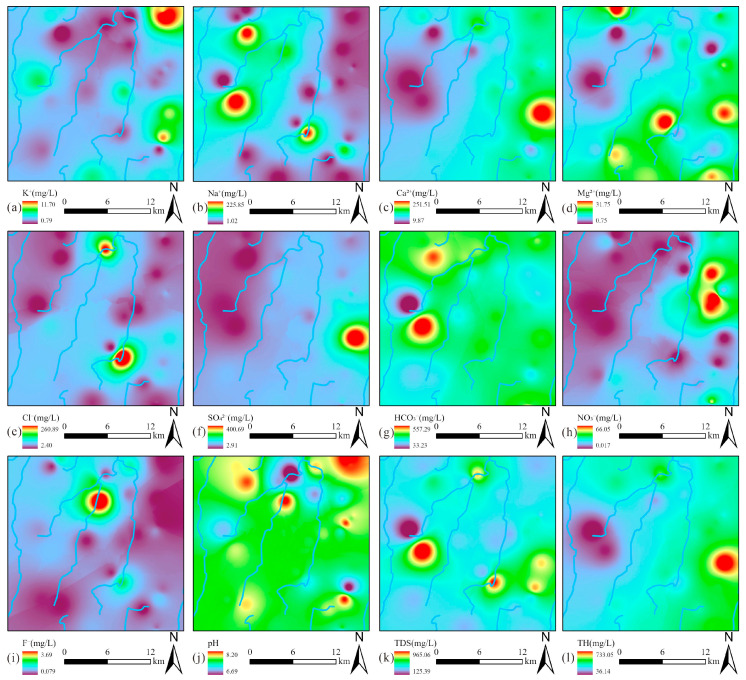
Spatial distribution maps of hydrochemical components in groundwater: (**a**) K^+^; (**b**) Na^+^; (**c**) Ca^2+^; (**d**) Mg^2+^; (**e**) Cl^−^; (**f**) SO_4_^2−^; (**g**) HCO_3_^−^; (**h**) NO_3_^−^; (**i**) F^−^; (**j**) pH; (**k**) TDS; (**l**) TH.

The spatial distribution characteristics of hydrochemical parameters were systematically analyzed through interpolation mapping (Figure 4). Notably, Na^+^ and Cl^−^ displayed strongly correlated distribution patterns, with their elevated concentrations predominantly clustered in the central valley area (Figure 4b,e), suggesting the same sources. In contrast, Ca^2+^, Mg^2+^, and SO_4_^2−^ exhibited remarkably similar spatial distributions, with their peak concentrations concentrated along the proper ridge (Figure 4c–f), indicating the same sources. The spatial distribution of HCO_3_^−^ showed a contrasting pattern, with elevated concentrations localized in the western left ridge region (Figure 4g). Of particular environmental concern, NO_3_^−^ contamination hotspots were identified in the northern sector of the proper ridge (Figure 4h). Meanwhile, elevated F^−^ concentrations formed a discrete anomaly in the north of the valley (Figure 4i). Notably, the spatial concentration distribution of Ca^2+^ was highly similar to that of TDS and TH, indicating that Ca^2+^ was the dominant ion controlling TDS and TH.

### 4.3. Natural Factors Affecting Groundwater Hydrochemistry

**Figure 5:**
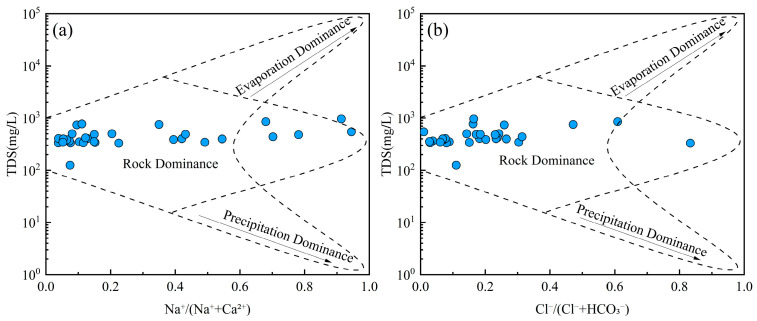
Gibbs diagrams of groundwater. (**a**) TDS vs. Na^+^/(Na^+^ + Ca^2+^); (**b**) TDS vs. Cl^−^/(Cl^−^ + HCO_3_^−^).

Groundwater chemistry is primarily governed by three natural processes: evaporation, rock weathering, and precipitation [62]. Gibbs diagrams are widely used to evaluate the dominant mechanism influencing the hydrochemical composition of groundwater [63]. All groundwater samples exhibited low TDS values (ranging from 100 to 1000 mg/L) and relatively low Na^+^/(Na^+^ + Ca^2+^) and Cl^−^/(Cl^−^ + HCO_3_^−^) ratios (Figure 5). These patterns suggest that the hydrochemical characteristics of groundwater were predominantly controlled by water–rock interactions rather than evaporation or atmospheric precipitation inputs. Groundwater is involved in various geological processes and ecological-environmental processes. The interaction between groundwater and rock is the driving force for the evolution of the near-surface environment [64]. The migration of groundwater solutes occurring in the water–rock system greatly affects the evolution of the groundwater environment [65].

**Figure 6:**
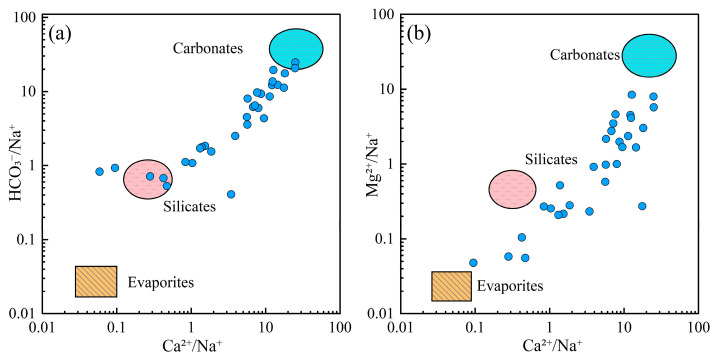
Gaillardet diagrams of groundwater. (**a**) HCO_3_^−^/Na^+^ vs. Ca^2+^/Na^+^; (**b**) Mg^2+^/Na^+^ vs. Ca^2+^/Na^+^.

To further elucidate the role of lithological influences on groundwater chemistry, Gaillardet diagrams were used to distinguish the contribution of different rock types (Figure 6). The majority of groundwater samples were situated around the carbonate end-member, indicating that carbonate rock dissolution was the primary mechanism controlling the chemical composition of groundwater in the region. A smaller number of samples aligned with the silicate weathering end-member, suggesting that silicate weathering also contributed to groundwater chemistry.

Previous studies have demonstrated that ionic ratios in groundwater are effective tools for identifying water–rock interactions within aquifer systems [66,67]. By analyzing various ionic ratios, it is possible to determine the sources of hydrochemical compositions, mineral weathering processes, and the influence of exogenous contamination.

**Figure 7:**
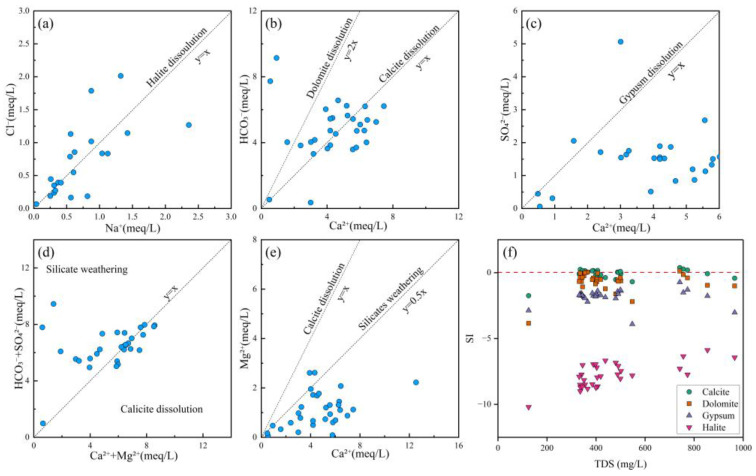
Ion ratio plots and SI vs. TDS plots of groundwater. (**a**) Cl^−^ vs. Na^+^; (**b**) HCO_3_^−^ vs. Ca^2+^; (**c**) SO_4_^2−^ vs. Ca^2+^; (**d**) HCO_3_^−^ + SO_4_^2−^ vs. Ca^2+^ + Mg^2+^; (**e**) Mg^2+^ vs. Ca^2+^; (**f**) SI vs. TDS.

The correlation between Na^+^ and Cl^−^ is commonly used to identify the influence of halite dissolution on hydrochemical composition [68]. When Na^+^ and Cl^−^ originate from halite dissolution, the Cl^−^/Na^+^ molar ratio equals 1, as represented by Equation (10). As shown in Figure 7a, some water samples exhibited Cl^−^/Na^+^ ratios near 1, indicating that Na^+^ was likely derived from halite dissolving. Moreover, some samples were located away from this line, meaning that human activities, silicate weathering, and cation exchange processes need to be further considered.

The correlation between Ca^2+^ and HCO_3_^−^ reflects dissolution processes of carbonate minerals such as calcite (CaCO_3_) and dolomite (CaMg(CO_3_)_2_) [69], as represented by Equations (11) and (12). As illustrated in Figure 7b, most water samples were plotted on both sides of the calcite dissolution line, indicating that the hydrochemical composition of groundwater in the study area was predominantly controlled by carbonate rock dissolution. Furthermore, a few samples clustered near the dolomite dissolution line, suggesting a localized influence from dolomite dissolution in certain zones.

The Ca^2+^/SO_4_^2−^ ratio serves as an effective indicator for assessing the dissolution contribution of gypsum (CaSO_4_·2H_2_O) [23], as expressed in Equation (13). As demonstrated in Figure 7c, most groundwater samples in the study area were plotted below the gypsum dissolution line, indicating limited hydrochemical contribution from sulfate mineral dissolution. However, a distinct subset of samples exhibited elevated SO_4_^2−^ concentrations, potentially attributable to anthropogenic inputs from sulfur-containing industrial wastewater and localized weathering of sulfate-bearing minerals.

The (HCO_3_^−^ + SO_4_^2−^)/(Ca^2+^ + Mg^2+^) ratio provides diagnostic insights for distinguishing between calcite dissolution and silicate weathering processes in water samples [70]. As evidenced in Figure 7d, most samples in the study area clustered along both sides of the calcite dissolution line, demonstrating that Ca^2+^ and Mg^2+^ primarily originated from carbonate mineral dissolution. Notably, a minor proportion of samples were plotted within the silicate weathering endmember zone, suggesting potential influence from weathering of silicate minerals (e.g., feldspars or pyroxenes) in localized areas.

The calcium–magnesium coefficient (Ca^2+^/Mg^2+^ ratio) was a robust hydrogeochemical indicator for evaluating water–rock interaction intensity and discriminating ion sources between carbonate and silicate mineral origins [71]. Most samples were below the line of 1:1, indicating that carbonate and silicate mineral dissolution contributed to Ca^2+^ and Mg^2+^.

(10) $$\mathrm{NaCl}\to {\mathrm{Na}}^{+}+{\mathrm{Cl}}^{-}$$

(11) $${\mathrm{CaCO}}_{3}\left(\mathrm{Calcite}\right)+H_{2}{\mathrm{CO}}_{3}\to {\mathrm{Ca}}^{2+}+2{\mathrm{HCO}}_{3}^{-}$$

(12) $$\mathrm{CaMg}{\left({\mathrm{CO}}_{3}\right)}_{2}\left(\mathrm{Dolomite}\right)+2H_{2}{\mathrm{CO}}_{3}\to {\mathrm{Ca}}^{2+}+{\mathrm{Mg}}^{2+}+4{\mathrm{HCO}}_{3}^{-}$$

(13) $${\mathrm{CaSO}}_{4}\cdot {2H}_{2}O\;\left(\mathrm{Gypsum}\right)\to {\mathrm{Ca}}^{2+}+{\mathrm{SO}}_{4}^{2-}+2H_{2}O$$

The Saturation Index (SI) serves as a fundamental geochemical parameter for evaluating the dissolution–precipitation equilibrium of specific minerals in groundwater and surface water systems, while simultaneously revealing potential sources of dissolved ions [72]. This study employed the PHREEQC 3.7.3 software package to systematically calculate and analyze mineral saturation indices (SI) for groundwater samples across the study area. The thermodynamic calculations for key minerals (dolomite, calcite, gypsum, and halite) were performed according to Equation (14), which rigorously accounted for aqueous speciation and mineral solubility equilibria.

(14) $$SI=\log\frac{IAP}{K_{sp}}$$

where *IAP* denotes the ion activity product in aqueous solutions, while *K_sp_* represents the temperature-specific solubility product constant of minerals.

An *SI* value below 0 indicated that the mineral was undersaturated, *SI* = 0 suggested a relatively equilibrium state, and an *SI* value above 0 signified that the mineral was in a supersaturated state [72]. In Figure 7f, the *SI* values of calcite (CaCO_3_) and dolomite (CaMg(CO_3_)_2_) were near 0, indicating that the dissolution of carbonate rocks was in equilibrium. In contrast, the *SI* values (<0) of gypsum (CaSO_4_·2H_2_O) and halite (NaCl) exhibited an undersaturated state. Therefore, the dissolution of sulfates and halite constituted the primary origin of the related ions, while the dissolution of carbonate rocks also contributed significantly to the relevant chemical composition.

**Figure 8:**
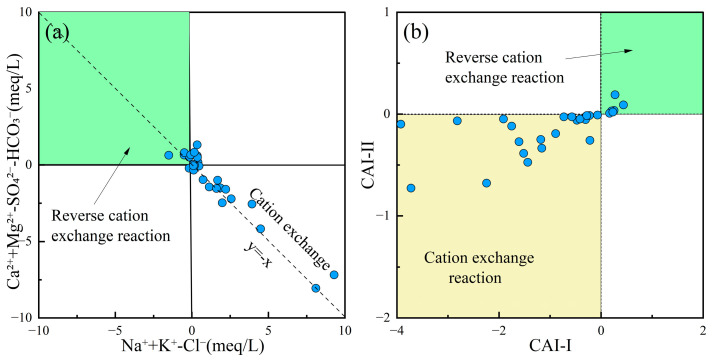
Cation exchange reaction plots of groundwater. (**a**) Ca^2+^ + Mg^2+^-SO_4_^2−^-HCO_3_^−^ vs. Na^+^ + K^+^-Cl^−^; (**b**) CAI-II vs. CAI-I.

Cation exchange often leads to variations in Na^+^, K^+^, Ca^2+^, and Mg^2+^ concentrations in groundwater, causing its chemical composition to deviate from the mineral dissolution-precipitation equilibrium state. The correlation between (Na^+^ + K^+^)–Cl^−^ and (Ca^2+^ + Mg^2+^)–(HCO_3_^−^ + SO_4_^2−^) could reflect cation exchange processes in groundwater [73]. Additionally, the Chloro-Alkaline Indices (CAI-I, Equation (7); CAI-II, Equation (8)) could determine whether cation exchange is direct or reverse, verifying the cation exchange processes occurring in the water [74]. Figure 8a,b show that most groundwater samples in the study area were distributed uniformly along the cation exchange line, indicating that the groundwater chemistry underwent direct cation exchange with clay minerals in the surrounding aquifer. This process resulted in the replacement of Ca^2+^ and Mg^2+^ by Na^+^ in the groundwater.

(15) $$\;\mathrm{CAI}-I=\frac{{\mathrm{Cl}}^{-}-\left({\mathrm{Na}}^{+}+K^{+}\right)}{{\mathrm{Cl}}^{-}}$$

(16) $$\mathrm{CAI}-\mathrm{II}=\frac{{\mathrm{Cl}}^{-}-\left({\mathrm{Na}}^{+}+K^{+}\right)}{{\mathrm{HCO}}_{3}^{-}+{\mathrm{SO}}_{4}^{2-}+{\mathrm{CO}}_{3}^{2-}+{\mathrm{NO}}_{3}^{-}}$$

### 4.4. Anthropogenic Factors Affecting Groundwater Hydrochemistry

**Figure 9:**
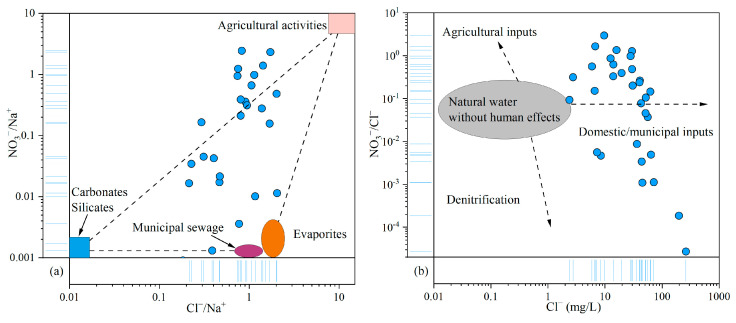
Relationship plots of NO_3_^−^, Cl^−^, and Na^+^ in groundwater: (**a**) NO_3_^−^/Na^+^ vs. Cl^−^/Na^+^; (**b**) NO_3_^−^/Cl^−^ vs. Cl^−^.

As discussed above, NO_3_^−^ was identified as the primary pollutant in the groundwater of the study area. Given the relative stability and conservative behavior of Cl^−^ in groundwater, it is commonly used as a tracer for identifying the sources of NO_3_^−^ contamination [75]. The relationship plot of NO_3_^−^/Na^+^ and Cl^−^/Na^+^ revealed that agricultural activities and municipal sewage contributed significantly to the NO_3_^−^ levels in groundwater (Figure 9a). The NO_3_^−^/Cl^−^ ratio typically ranges from 0.05 to 0.22 in natural water. Ratios exceeding this range generally indicate anthropogenic influences, including inputs from agricultural fertilizers, domestic wastewater, and denitrification processes. In this study, NO_3_^−^/Cl^−^ ratios varied widely, from 2.72 × 10^−5^ to 2.97, far exceeding the natural baseline and reflecting human impacts. The relationship between NO_3_^−^/Cl^−^ and Cl^−^ concentrations further supported the conclusion that domestic and municipal waste were the primary sources of NO_3_^−^, with additional contributions from agricultural activities (Figure 9b).

**Figure 10:**
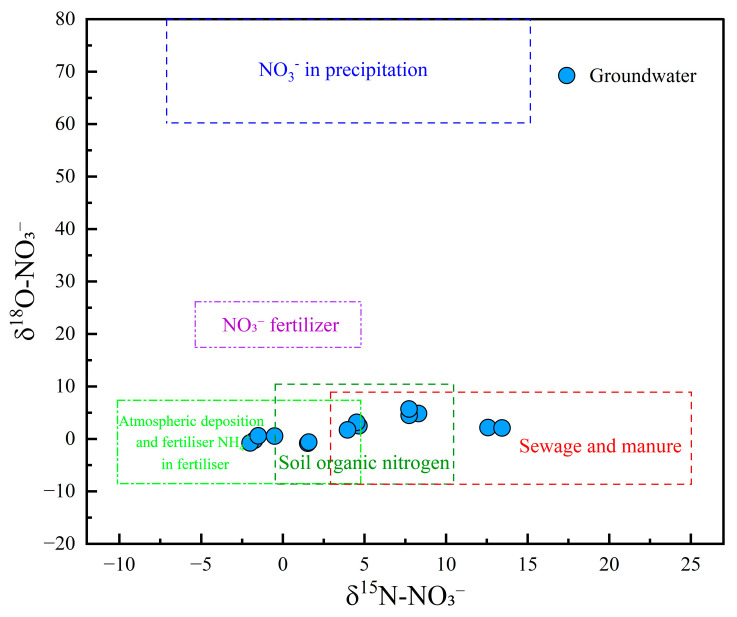
Cross plot of δ^18^O-NO_3_ and δ^15^N-NO_3_ in groundwater.

The dual isotopes of NO_3_^−^ (δ^15^N-NO_3_^−^ and δ^18^O-NO_3_^−^) are recognized as conservative tracers that provided valuable insights into the multiple sources of NO_3_^−^ in groundwater [76]. In this study, δ^15^N-NO_3_^−^ values ranged from −0.52‰ to 8.31‰, with a mean value of 5.07‰, while δ^18^O-NO_3_^−^ ranged from 0.56‰ to 5.72‰, with a mean of 2.20‰. As illustrated in the δ^15^N–δ^18^O plot (Figure 10), no groundwater samples were located in the region associated with atmospheric precipitation and NO_3_^−^ fertilizers, suggesting that these sources had limited influence on NO_3_^−^ levels. Two samples were plotted in the overlapping area of NH_4_^+^ fertilizer and soil organic nitrogen, indicating a mixed contribution from both sources. Three samples were positioned in the mixed soil organic nitrogen and sewage/manure region, suggesting combined inputs from natural and anthropogenic nitrogen sources. Furthermore, three groundwater samples fell within the mixed domain of NH_4_^+^ fertilizer, soil organic nitrogen, and sewage and manure, indicating that all three sources jointly contributed to NO_3_^−^ pollution. Notably, three and two samples were located in the exclusive zones of NH_4_^+^ fertilizer and sewage and manure, respectively, implying that these sources were singularly responsible for the contamination in those samples. These results underscored the complex and variable origins of NO_3_^−^ in the study area, with contributions from soil organic nitrogen, agricultural practices (e.g., fertilizer application), and anthropogenic waste (e.g., sewage discharge and manure accumulation).

### 4.5. The Impact of Land Use Types on Nitrate Concentrations

**Figure 11:**
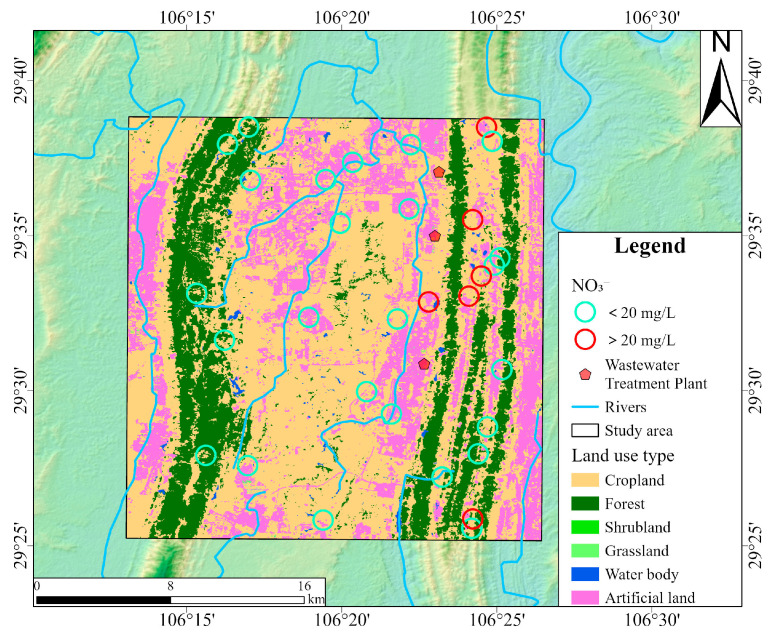
The land use map of the study area and the locations of groundwater samples with NO_3_^−^ concentrations.

Land use types exerted differential impacts on groundwater quality, particularly concerning NO_3_^−^ concentrations [77]. As shown in Figure 11, red circles indicate groundwater sampling sites where NO_3_^−^ concentrations exceeded the Chinese drinking water guideline (20 mg/L), while green circles represent compliant sites. Elevated NO_3_^−^ concentrations were predominantly distributed in the northern and southern parts of the proper ridge, where land use was primarily characterized by cropland and artificial surfaces. This spatial pattern aligned well with the dual isotope evidence presented earlier. This supports the conclusion that NO_3_^−^ in groundwater mainly originated from the mineralization of soil organic nitrogen, NH_4_^+^ fertilizer, and sewage and manure infiltration. In cropland areas, nitrogen fertilizers dissolve in water and are subsequently absorbed by crops in the form of NH_4_^+^ in the soil. Excess nitrogen not utilized by plants could be oxidized to NO_3_^−^ and leached into the groundwater system. In rural settlements, direct discharge of domestic sewage and improper livestock manure storage could further exacerbate NO_3_^−^ pollution through surface runoff and infiltration. Moreover, three wastewater treatment plants are situated in the study area. During heavy rainfall, substantial stormwater infiltrates the sewer system, combining with wastewater and exceeding the treatment capacity of the wastewater treatment plants. This hydraulic overload leads to frequent combined sewer overflows, indicating insufficient infrastructure development, particularly in the sewage pipe network and related facilities. In contrast, lower NO_3_^−^ concentrations were observed in the left ridge, where grassland was the dominant land cover. Field investigations revealed extremely minimal levels of anthropogenic disturbance in grassland areas. This reflected the nitrogen fixation capacity of grassland ecosystems [78]. The spatial correlation analysis between NO_3_^−^ concentrations and land use types revealed a transparent gradient in NO_3_^−^ pollution potential: artificial surfaces > cropland > grassland, underscoring the importance of land management in mitigating NO_3_^−^ contamination risks in groundwater systems.

### 4.6. Drinking Water Quality Assessment

**Figure 12:**
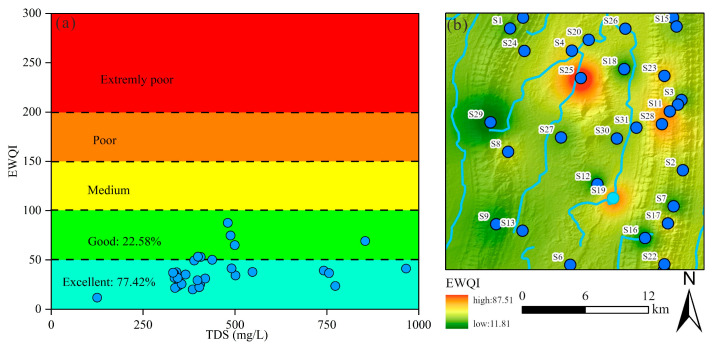
The drinking water quality of groundwater: (**a**) EWQI vs. TDS plot; (**b**) the spatial distribution map of EWQI in study area.

In this study, the EWQI was utilized to assess the suitability of groundwater for drinking purposes. Previous research has established that EWQI values below 100 indicate water of acceptable quality for human consumption. The groundwater quality evaluation incorporated 12 hydrochemical parameters: K^+^, Na^+^, Ca^2^^+^, Mg^2^^+^, Cl^−^, SO_4_*^2^*^−^, HCO_3_^−^, NO_3_^−^, F^−^, pH, TDS, and TH. Among these parameters, NO_3_^−^ (weight = 0.17), F^−^ (0.17), Cl^−^ (0.16), and Na^+^ (0.15) were assigned higher weighting coefficients, identifying them as the primary factors influencing groundwater quality for drinking purposes (Table 2). The calculated EWQI values across all samples ranged from 11.81 to 87.51, with a mean value of 38.83. Based on the EWQI classification scheme, all sampled groundwater was deemed suitable for drinking. Specifically, 77.42% of samples were classified as excellent quality, while the remaining 22.58% were considered good quality (Figure 12a). These results demonstrate that the studied groundwater sources met established drinking water standards.

A Geographic Information System (GIS) approach was employed further to analyze the geospatial distribution of drinking water quality. As illustrated in Figure 12b, elevated EWQI values were observed in the northern and eastern regions, attributable to the influence of adjacent croplands (Figure 12). Although the overall groundwater quality ranges from good to excellent, these spatial patterns suggest that anthropogenic activities, particularly agricultural practices, may contribute to localized water quality degradation. Consequently, targeted monitoring and management strategies should be implemented to mitigate potential contamination risks.

## 5. Conclusions

In this study, a total of 31 groundwater samples were collected from a typical urban area in southwestern China. This study elucidated the hydrochemical processes, sources of NO_3_^−^ pollution, the impact of land use on NO_3_^−^ levels, and drinking water safety. The key conclusions are summarized as follows:

(1) The groundwater was neutral to slightly alkaline in nature, and the groundwater type was Ca-HCO_3_. The cation concentrations exhibited the following order: Ca^2+^ > Na^+^ > Mg^2+^ > K^+^, while the anion concentrations followed this order: HCO_3_^−^ > SO_4_^2−^ > Cl^−^ >NO_3_^−^ > F^−^. The Gibbs diagram, ion ratio, and mineral saturation index results indicated that the dissolution of carbonates and the weathering of silicates were the primary controlling factors for hydrochemical characteristics.

(2) The NO_3_^−^ concentrations ranged from 0.01 mg/L to 66.08 mg/L, with 19.35% of the samples exceeding the Chinese permissible limits. The ion ratio relationship of NO_3_^−^, Na^+^, and Cl^−^ showed that NO_3_^−^ pollution originated from agricultural activities, sewage discharge, and manure. Further analysis of the dual isotopes δ^15^N and δ^18^O revealed that the high-concentration NO_3_^−^ pollution in groundwater mainly resulted from NH_4_^+^ fertilizer, soil organic nitrogen, and domestic sewage and manure.

(3) The land use map revealed that elevated NO_3_^−^ concentrations were found in the agricultural and artificial surface areas near the proper ridge, while low NO_3_^−^ concentrations were found in the grassland areas. Thus, the spatial distribution of NO_3_^−^ correlated strongly with land use types. In agricultural regions, unabsorbed NH_4_^+^ from nitrogen fertilizer entered the groundwater through precipitation and irrigation water infiltration. In artificial surface areas, the discharge of domestic sewage and the disposal of livestock manure contributed to NO_3_^−^ pollution. In the grassland areas situated on the ridge, the nitrogen fixation capacity of the grassland ecosystem contributed to the low levels of NO_3_^−^ concentration.

(4) The EWQI method indicated that 77.42% of samples were classified as excellent quality, while 22.58% were categorized as good quality. Therefore, all the groundwater was suitable for drinking. The relatively elevated EWQI values, caused by NO_3_^−^ and F^−^, were situated in the northern and eastern regions of the study area.

## Figures and Tables

**Table 1:** Statistical analysis of hydrochemical parameters in 31 groundwater samples (units of all parameters are mg/L, except pH).

Parameters	Min	Max	Mean	SD	CV (%)	Limit	% of SEL
pH	6.69	8.20	7.63	0.36	5.00	6.5–8.5 ^a^	0
TH	36.03	733.16	318.56	118.06	37.00	450 ^a^	3.22
TDS	125.00	965.55	461.54	176.54	38.00	1000 ^a^	0
K^+^	0.79	11.73	3.59	2.40	67.00	-	-
Na^+^	0.91	225.98	54.73	63.74	116.00	200 ^a^	3.22
Ca^2+^	9.85	251.55	93.47	46.46	50.00	400 ^b^	0
Mg^2+^	0.75	31.77	13.60	8.30	61.00	50 ^b^	0
Cl^−^	2.37	261	42.12	53.50	127.00	250 ^a^	3.22
SO_4_^2−^	2.90	400.77	84.09	66.42	79.00	250 ^a^	3.22
HCO_3_^−^	32.94	557.57	303.18	91.24	30.00	-	-
NO_3_^−^	0.01	66.08	13.56	16.46	121.00	20 ^a^	19.35
F^−^	0.08	3.69	0.61	0.73	119.00	1 ^a^	16.13

^a^ Standard for groundwater quality (GB/T 14848−2017). ^b^ Standards for drinking water quality (GB 5749−2022).

**Table 2:** Relative weights of each hydrochemical parameter.

Parameters	K^+^	Na^+^	Ca^2+^	Mg^2+^	Cl^−^	SO_4_^2−^	HCO_3_^−^	NO_3_^−^	F^−^	TDS	TH	pH
*w_j_*	0.083	0.15	0.045	0.059	0.16	0.063	0.018	0.17	0.17	0.034	0.026	0.024

## Data Availability

The data presented in this study are available upon request from the corresponding author.

## References

[1] Scanlon B.R., Fakhreddine S., Rateb A., De Graaf I., Famiglietti J., Gleeson T., Grafton R.Q., Jobbagy E., Kebede S., Kolusu S.R. (2023). Author Correction: Global Water Resources and the Role of Groundwater in a Resilient Water Future. Nat. Rev. Earth Environ..

[2] Burri N.M., Weatherl R., Moeck C., Schirmer M. (2019). A Review of Threats to Groundwater Quality in the Anthropocene. Sci. Total Environ..

[3] Wei D., Yang S., Zou L., Torres-Martínez J.A., Zheng Y., Hu Q., Zhang Y. (2025). Appraisal of Potential Toxic Elements Pollution, Sources Apportionment, and Health Risks in Groundwater from a Coastal Area of SE China. J. Environ. Manag..

[4] Sforzi L., Sarti C., Santini S., Martellini T., Cincinelli A. (2024). Global Status, Risk Assessment, and Knowledge Gaps of Microplastics in Groundwater: A Bibliometric Analysis. Groundw. Sustain. Dev..

[5] Wick K., Heumesser C., Schmid E. (2012). Groundwater Nitrate Contamination: Factors and Indicators. J. Environ. Manag..

[6] Mahvi A.H., Nouri J., Babaei A.A., Nabizadeh R. (2005). Agricultural Activities Impact on Groundwater Nitrate Pollution. Int. J. Environ. Sci. Technol..

[7] McLay C.D.A., Dragten R., Sparling G., Selvarajah N. (2001). Predicting Groundwater Nitrate Concentrations in a Region of Mixed Agricultural Land Use: A Comparison of Three Approaches. Environ. Pollut..

[8] Selvam S., Nath A.V., Roy P.D., Jesuraja K., Muthukumar P. (2023). Evaluation of Groundwater for Nitrate and Fluoride in Alappuzha Region from the Southwestern Coast of India and Associated Health Risks. Environ. Res..

[9] Zhang X., Xu Z., Sun X., Dong W., Ballantine D. (2013). Nitrate in Shallow Groundwater in Typical Agricultural and Forest Ecosystems in China, 2004–2010. J. Environ. Sci..

[10] Bijay-Singh, Craswell E. (2021). Fertilizers and Nitrate Pollution of Surface and Ground Water: An Increasingly Pervasive Global Problem. SN Appl. Sci..

[11] Peña-Haro S., Llopis-Albert C., Pulido-Velazquez M., Pulido-Velazquez D. (2010). Fertilizer Standards for Controlling Groundwater Nitrate Pollution from Agriculture: El Salobral-Los Llanos Case Study, Spain. J. Hydrol..

[12] Singh B., Singh Y., Sekhon G.S. (1995). Fertilizer-N Use Efficiency and Nitrate Pollution of Groundwater in Developing Countries. J. Contam. Hydrol..

[13] Chaudhary I.J., Chauhan R., Kale S.S., Gosavi S., Rathore D., Dwivedi V., Singh S., Yadav V.K. (2025). Groundwater Nitrate Contamination and Its Effect on Human Health: A Review. Water Conserv. Sci. Eng..

[14] Rajan M., Karunanidhi D., Jaya J., Preethi B., Subramani T., Aravinthasamy P. (2024). A Comprehensive Review on Human Health Hazards Due to Groundwater Contamination: A Global Perspective. Phys. Chem. Earth Parts A/B/C.

[15] Karunanidhi D., Aravinthasamy P., Subramani T., Kumar M. (2021). Human Health Risks Associated with Multipath Exposure of Groundwater Nitrate and Environmental Friendly Actions for Quality Improvement and Sustainable Management: A Case Study from Texvalley (Tiruppur Region) of India. Chemosphere.

[16] Zhang Y., Yan Y., Yao R., Wei D., Huang X., Luo M., Wei C., Chen S., Yang C. (2024). Natural Background Levels, Source Apportionment and Health Risks of Potentially Toxic Elements in Groundwater of Highly Urbanized Area. Sci. Total Environ..

[17] Wang Y., Li R., Wu X., Yan Y., Wei C., Luo M., Xiao Y., Zhang Y. (2023). Evaluation of Groundwater Quality for Drinking and Irrigation Purposes Using GIS-Based IWQI, EWQI and HHR Model. Water.

[18] He S., Li P., Su F., Wang D., Ren X. (2022). Identification and Apportionment of Shallow Groundwater Nitrate Pollution in Weining Plain, Northwest China, Using Hydrochemical Indices, Nitrate Stable Isotopes, and the New Bayesian Stable Isotope Mixing Model (MixSIAR). Environ. Pollut..

[19] Liu J., Peng Y., Li C., Gao Z., Chen S. (2021). Characterization of the Hydrochemistry of Water Resources of the Weibei Plain, Northern China, as Well as an Assessment of the Risk of High Groundwater Nitrate Levels to Human Health. Environ. Pollut..

[20] Cao M., Hu A., Gad M., Adyari B., Qin D., Zhang L., Sun Q., Yu C.-P. (2022). Domestic Wastewater Causes Nitrate Pollution in an Agricultural Watershed, China. Sci. Total Environ..

[21] Zhang Y., Li F., Zhang Q., Li J., Liu Q. (2014). Tracing Nitrate Pollution Sources and Transformation in Surface- and Ground-Waters Using Environmental Isotopes. Sci. Total Environ..

[22] Richa A., Touil S., Fizir M. (2022). Recent Advances in the Source Identification and Remediation Techniques of Nitrate Contaminated Groundwater: A Review. J. Environ. Manag..

[23] Yang Q., Wang L., Ma H., Yu K., Martín J.D. (2016). Hydrochemical Characterization and Pollution Sources Identification of Groundwater in Salawusu Aquifer System of Ordos Basin, China. Environ. Pollut..

[24] Jin Z., Pan Z., Jin M., Li F., Wan Y., Gu B. (2012). Determination of Nitrate Contamination Sources Using Isotopic and Chemical Indicators in an Agricultural Region in China. Agric. Ecosyst. Environ..

[25] Jamshidi A., Morovati M., Golbini Mofrad M.M., Panahandeh M., Soleimani H., Abdolahpour Alamdari H. (2021). Water Quality Evaluation and Non-Cariogenic Risk Assessment of Exposure to Nitrate in Groundwater Resources of Kamyaran, Iran: Spatial Distribution, Monte-Carlo Simulation, and Sensitivity Analysis. J. Environ. Health Sci. Eng..

[26] Adimalla N., Qian H. (2019). Groundwater Quality Evaluation Using Water Quality Index (WQI) for Drinking Purposes and Human Health Risk (HHR) Assessment in an Agricultural Region of Nanganur, South India. Ecotoxicol. Environ. Saf..

[27] Ram A., Tiwari S.K., Pandey H.K., Chaurasia A.K., Singh S., Singh Y.V. (2021). Groundwater Quality Assessment Using Water Quality Index (WQI) under GIS Framework. Appl. Water Sci..

[28] Xie Z., Liu W., Chen S., Yao R., Yang C., Zhang X., Li J., Wang Y., Zhang Y. (2025). Machine Learning Approaches to Identify Hydrochemical Processes and Predict Drinking Water Quality for Groundwater Environment in a Metropolis. J. Hydrol. Reg. Stud..

[29] Yao R., Zhang Y., Yan Y., Wu X., Uddin M.G., Wei D., Huang X., Tang L. (2024). Natural Background Level, Source Apportionment and Health Risk Assessment of Potentially Toxic Elements in Multi-Layer Aquifers of Arid Area in Northwest China. J. Hazard. Mater..

[30] Su K., Wang Q., Li L., Cao R., Xi Y. (2022). Water Quality Assessment of Lugu Lake Based on Nemerow Pollution Index Method. Sci. Rep..

[31] Maskooni E., Naseri-Rad M., Berndtsson R., Nakagawa K. (2020). Use of Heavy Metal Content and Modified Water Quality Index to Assess Groundwater Quality in a Semiarid Area. Water.

[32] Sadat-Noori S.M., Ebrahimi K., Liaghat A.M. (2014). Groundwater Quality Assessment Using the Water Quality Index and GIS in Saveh-Nobaran Aquifer, Iran. Environ. Earth Sci..

[33] Boateng T.K., Opoku F., Acquaah S.O., Akoto O. (2016). Groundwater Quality Assessment Using Statistical Approach and Water Quality Index in Ejisu-Juaben Municipality, Ghana. Environ. Earth Sci..

[34] Bouteraa O., Mebarki A., Bouaicha F., Nouaceur Z., Laignel B. (2019). Groundwater Quality Assessment Using Multivariate Analysis, Geostatistical Modeling, and Water Quality Index (WQI): A Case of Study in the Boumerzoug-El Khroub Valley of Northeast Algeria. Acta Geochim..

[35] Kumi, Michael, Anku, Wilson W., Antwi, Yeboah B., Penny, Govender P. (2023). Evaluation of the Suitability of Integrated Bone Char- and Biochar-Treated Groundwater for Drinking Using Single-Factor, Nemerow, and Heavy Metal Pollution Indexes. Environ. Monit. Assess..

[36] Chen R.-H., Li F.-P., Zhang H.-P., Jiang Y., Mao L.-C., Wu L.-L., Chen L. (2016). Comparative Analysis of Water Quality and Toxicity Assessment Methods for Urban Highway Runoff. Sci. Total Environ..

[37] Deng J., Yang G., Yan X., Du J., Tang Q., Yu C., Pu S. (2024). Quality Evaluation and Health Risk Assessment of Karst Groundwater in Southwest China. Sci. Total Environ..

[38] Rupias O.J.B., Pereira S.Y., De Abreu A.E.S. (2021). Hydrogeochemistry and Groundwater Quality Assessment Using the Water Quality Index and Heavy-Metal Pollution Index in the Alluvial Plain of Atibaia River- Campinas/SP, Brazil. Groundw. Sustain. Dev..

[39] Abdessamed D., Jodar-Abellan A., Ghoneim S.S.M., Almaliki A., Hussein E.E., Pardo M.Á. (2023). Groundwater Quality Assessment for Sustainable Human Consumption in Arid Areas Based on GIS and Water Quality Index in the Watershed of Ain Sefra (SW of Algeria). Environ. Earth Sci..

[40] Seifi A., Dehghani M., Singh V.P. (2020). Uncertainty Analysis of Water Quality Index (WQI) for Groundwater Quality Evaluation: Application of Monte-Carlo Method for Weight Allocation. Ecol. Indic..

[41] Cui H., Duan L., Pan H., Liu T. (2025). Geochemical Pattern, Quality and Driving Forces of Multi-Layer Groundwater in a High-Capacity Mining Area Basin: A Comprehensive Analysis Based on the Interweaving of Multiple Factors. J. Hydrol..

[42] Choudhary S., Subba Rao N., Chaudhary M., Das R. (2024). Assessing Sources of Groundwater Quality and Health Risks Using Graphical, Multivariate, and Index Techniques from a Part of Rajasthan, India. Groundw. Sustain. Dev..

[43] Du J., Jia C., Ding Y., Yang X., Feng K., Wei M. (2025). Advancing Wetland Groundwater Pollution Zoning: A Novel Integration of Monte Carlo Health Risk Modeling and Machine Learning. J. Hazard. Mater..

[44] Dogramaci S., Skrzypek G., Dodson W., Grierson P.F. (2012). Stable Isotope and Hydrochemical Evolution of Groundwater in the Semi-Arid Hamersley Basin of Subtropical Northwest Australia. J. Hydrol..

[45] Ujević Bošnjak M., Capak K., Jazbec A., Casiot C., Sipos L., Poljak V., Dadić Ž. (2012). Hydrochemical Characterization of Arsenic Contaminated Alluvial Aquifers in Eastern Croatia Using Multivariate Statistical Techniques and Arsenic Risk Assessment. Sci. Total Environ..

[46] Fu C., Li X., Ma J., Liu L., Gao M., Bai Z. (2018). A Hydrochemistry and Multi-Isotopic Study of Groundwater Origin and Hydrochemical Evolution in the Middle Reaches of the Kuye River Basin. Appl. Geochem..

[47] Nath A.V., Sekar S., Roy P.D., Kamaraj J., Shukla S., Khan R. (2024). Drinking and Irrigation Quality and Pollution Assessments of the Groundwater Resources from Alappuzha in Kerala (India) through an Integrated Approach Using WQI and GIS. J. Geochem. Explor..

[48] Krishan G., Kumar M., Rao M.S., Garg R., Yadav B.K., Kansal M.L., Singh S., Bradley A., Muste M., Sharma L.M. (2023). Integrated Approach for the Investigation of Groundwater Quality through Hydrochemistry and Water Quality Index (WQI). Urban Clim..

[49] Yao R., Xu J., Zhou Y., Li S., Su J., Yan Y., Gan Y., Luo M., Zhang Y. (2025). Hydrochemical Evolution and Assessment of Groundwater Quality in an Intensively Agricultural Area: Case Study of Chengdu Plain, Southwestern China. Environ. Earth Sci..

[50] Ahmed S., Haque K.E., Moniruzzaman M., Suborna M.A.K., Anonna T., Bhuyian M.A.Q., Ahsan M.A., Khan A.H.A.N., Karim M.M., Kasem M.A. (2025). Hydrogeochemistry, Water Quality, and Potential Human Health Risk Assessment of Groundwater in a Drought-Prone Area, Bangladesh. Groundw. Sustain. Dev..

[51] Vesković J., Deršek-Timotić I., Lučić M., Miletić A., Đolić M., Ražić S., Onjia A. (2024). Entropy-Weighted Water Quality Index, Hydrogeochemistry, and Monte Carlo Simulation of Source-Specific Health Risks of Groundwater in the Morava River Plain (Serbia). Mar. Pollut. Bull..

[52] Rajan S., Nandimandalam J.R., Ram P. (2025). Hydrogeochemical Evolution of Spring Water in the Western Lower Himalayas: Seasonal Changes, Quality Assessment, and Health Risks. Groundw. Sustain. Dev..

[53] Nisar U.B., Rehman W.U., Saleem S., Taufail K., Rehman F.U., Farooq M., Ehsan S.A. (2024). Assessment of Water Quality Using Entropy-Weighted Quality Index, Statistical Methods and Electrical Resistivity Tomography, Moti Village, Northern Pakistan. J. Contam. Hydrol..

[54] Liu M. (2022). Response of Groundwater Chemical Characteristics to Land Use Types and Health Risk Assessment of Nitrate in Semi-Arid Areas: A Case Study of Shuangliao City, Northeast China. Ecotoxicol. Environ. Saf..

[55] Wang S., Zheng W., Currell M., Yang Y., Zhao H., Lv M. (2017). Relationship between Land-Use and Sources and Fate of Nitrate in Groundwater in a Typical Recharge Area of the North China Plain. Sci. Total Environ..

[56] Choi W.-J., Han G.-H., Lee S.-M., Lee G.-T., Yoon K.-S., Choi S.-M., Ro H.-M. (2007). Impact of Land-Use Types on Nitrate Concentration and δ15N in Unconfined Groundwater in Rural Areas of Korea. Agric. Ecosyst. Environ..

[57] Iqbal J., Su C., Abbas H., Jiang J., Han Z., Baloch M.Y.J., Xie X. (2025). Prediction of Nitrate Concentration and the Impact of Land Use Types on Groundwater in the Nansi Lake Basin. J. Hazard. Mater..

[58] Zhang Y., Dai Y., Wang Y., Huang X., Xiao Y., Pei Q. (2021). Hydrochemistry, Quality and Potential Health Risk Appraisal of Nitrate Enriched Groundwater in the Nanchong Area, Southwestern China. Sci. Total Environ..

[59] Cui R., Chen A., Hu W., Fu B., Liu G., Zhang D. (2024). Appropriate Stoichiometric Ratios of Dissolved Organic Carbon and Nitrate Can Trigger a Transition in Nitrate Removal in Groundwater around Plateau Lakes, Southwest China. Sci. Total Environ..

[60] Piper A.M. (1944). A Graphic Procedure in the Geochemical Interpretation of Water-Analyses. Eos Trans. Am. Geophys. Union.

[61] (2017). Standard for Groundwater Quality.

[62] Mao M., Wang X., Zhu X. (2021). Hydrochemical Characteristics and Pollution Source Apportionment of the Groundwater in the East Foothill of the Taihang Mountains, Hebei Province. Environ. Earth Sci..

[63] Gibbs R.J. (1970). Mechanisms Controlling World Water Chemistry. Science.

[64] Samtio M.S., Hakro A.A.A.D., Jahangir T.M., Mastoi A.S., Lanjwani M.F., Rajper R.H., Lashari R.A., Agheem M.H., Noonari M.W. (2023). Impact of Rock-Water Interaction on Hydrogeochemical Characteristics of Groundwater: Using Multivariate Statistical, Water Quality Index and Irrigation Indices of Chachro Sub-District, Thar Desert, Sindh, Pakistan. Groundw. Sustain. Dev..

[65] Liu Z., Wang X., Wan X., Jia S., Mao B. (2024). Evolution Origin Analysis and Health Risk Assessment of Groundwater Environment in a Typical Mining Area: Insights from Water-Rock Interaction and Anthropogenic Activities. Environ. Res..

[66] Chowdhury P., Mukhopadhyay B.P., Bera A. (2022). Hydrochemical Assessment of Groundwater Suitability for Irrigation in the North-Eastern Blocks of Purulia District, India Using GIS and AHP Techniques. Phys. Chem. Earth Parts A/B/C.

[67] Adjéï Kouacou B., Anornu G., Adiaffi B., Gibrilla A. (2024). Hydrochemical Characteristics and Sources of Groundwater Pollution in Soubré and Gagnoa Counties, Côte d’Ivoire. Groundw. Sustain. Dev..

[68] Yu H., Gui H., Zhao H., Wang M., Li J., Fang H., Jiang Y., Zhang Y. (2020). Hydrochemical Characteristics and Water Quality Evaluation of Shallow Groundwater in Suxian Mining Area, Huaibei Coalfield, China. Int. J. Coal Sci. Technol..

[69] Wang S., Chen J., Zhang S., Zhang X., Chen D., Zhou J. (2023). Hydrochemical Evolution Characteristics, Controlling Factors, and High Nitrate Hazards of Shallow Groundwater in a Typical Agricultural Area of Nansi Lake Basin, North China. Environ. Res..

[70] Sheng D., Meng X., Wen X., Wu J., Yu H., Wu M., Zhou T. (2023). Hydrochemical Characteristics, Quality and Health Risk Assessment of Nitrate Enriched Coastal Groundwater in Northern China. J. Clean. Prod..

[71] Subba Rao N., Das R., Sahoo H.K., Gugulothu S. (2024). Hydrochemical Characterization and Water Quality Perspectives for Groundwater Management for Urban Development. Groundw. Sustain. Dev..

[72] Gao Y., Qian H., Ren W., Wang H., Liu F., Yang F. (2020). Hydrogeochemical Characterization and Quality Assessment of Groundwater Based on Integrated-Weight Water Quality Index in a Concentrated Urban Area. J. Clean. Prod..

[73] Fan W., Zhou J., Zheng J., Guo Y., Hu L., Shan R. (2024). Hydrochemical Characteristics, Control Factors and Health Risk Assessment of Groundwater in Typical Arid Region Hotan Area, Chinese Xinjiang. Environ. Pollut..

[74] Hao Q., Li Y., Xiao Y., Yang H., Zhang Y., Wang L., Liu K., Liu G., Wang J., Hu W. (2023). Hydrogeochemical Fingerprint, Driving Forces and Spatial Availability of Groundwater in a Coastal Plain, Southeast China. Urban Clim..

[75] Wang D., Li P., Yang N., Yang C., Zhou Y., Li J. (2023). Distribution, Sources and Main Controlling Factors of Nitrate in a Typical Intensive Agricultural Region, Northwestern China: Vertical Profile Perspectives. Environ. Res..

[76] Ding K., Zhang Y., Zhang H., Yu C., Li X., Zhang M., Zhang Z., Yang Y. (2024). Tracing Nitrate Origins and Transformation Processes in Groundwater of the Hohhot Basin’s Piedmont Strong Runoff Zone through Dual Isotopes and Hydro-Chemical Analysis. Sci. Total Environ..

[77] Zhang X., Gao S., Wu Q., Li F., Wu P., Wang Z., Wu J., Zeng J. (2023). Buffer Zone-Based Trace Elements Indicating the Impact of Human Activities on Karst Urban Groundwater. Environ. Res..

[78] Chen P., Ma J., Yue X., Zeng H., Wang C., Huang Q., Zhou Y., Zhang L. (2025). Nitrate Dynamics in Deep Soils of the Loess Plateau: Impact of Different Land Use Types. Ecol. Indic..

